# Migrants’ perceptions of aging in Denmark and attitudes towards remigration: findings from a qualitative study

**DOI:** 10.1186/s12913-015-0901-5

**Published:** 2015-06-07

**Authors:** Maria Kristiansen, Linnea Lue Kessing, Marie Norredam, Allan Krasnik

**Affiliations:** Center for Healthy Aging, and Research Centre for Migration, Ethnicity, and Health, Department of Public Health, Faculty of Health and Medical Sciences, University of Copenhagen, Øster Farimagsgade 5A, 1014 Copenhagen K, Denmark; Section of Immigrant Medicine, Department of Infectious Diseases, Copenhagen University Hospital, Hvidovre, Denmark; Centre for Healthy Aging, Department of Public Health, Faculty of Health and Medical Sciences, University of Copenhagen, Øster Farimagsgade 5A, 1014 Copenhagen K, Denmark

**Keywords:** Migrants, Europe, Aging, Healthcare services, Social services, Adaption, Return Migration

## Abstract

**Background:**

The increasing number of elderly migrants in Europe poses challenges for the organisation of healthcare and social services if these migrants do not remigrate to their countries of birth at old age. More insight into perceptions of aging among migrant women is needed to inform service delivery for culturally and linguistic diverse populations, yet few studies have explored this field. The aim of this study is to explore perceptions of aging among middle-aged migrant women, with emphasis on identifying factors shaping their decisions on whether to remigrate or stay in Denmark during old age.

**Methods:**

The study is based on 14 semi-structured interviews including a total of 29 migrant women residing in Copenhagen, Denmark. The women were born in Somalia, Turkey, India, Iran, Pakistan, or Middle Eastern countries. The majority of participants were middle-aged and had one or more chronic illnesses. The analysis was inspired by phenomenological methods and guided by theory on access to services, social relations, and belonging.

**Results:**

The results showed that the existence of chronic conditions requiring frequent use of medical care and the availability of high-quality healthcare in Denmark were important factors for the decision to spend one’s old age in Denmark rather than to remigrate to one’s country of origin. Similarly, availability of social services providing financial and tangible support for the elderly was perceived to be important during old age. For these middle-aged women, social ties to children and grandchildren in Denmark and feelings of belonging further nourished a wish to stay in Denmark rather than remigrating.

**Conclusions:**

Since the study suggests that elderly migrants will be utilising healthcare and social services in Denmark rather than returning to their countries of birth, these services should prepare for increased cultural and linguistic diversity among users. This could entail provision of translators, specific outreach programmes, and culturally adapted services to meet elderly from diverse linguistic, religious, and cultural backgrounds.

## Background

The increasing number of elderly individuals worldwide has resulted in growing demand for long-term care, with implications for the organisation and financing of healthcare and social services [1, 2]. Along with these demographic changes, migration flows have led to an increase in the number of migrant populations approaching old age, particularly in Europe [3, 4]. Elderly migrants may have different healthcare needs, for example due to different morbidity patterns and language barriers [5]. Furthermore, their expectations of family support and social care services for the elderly may also differ from those of the ethnic majority [6, 7]. The growing population of elderly migrants in Europe comprises both refugees and people who have come to Europe through voluntary migration. Socioeconomic and cultural background, number of years since immigration, possibility for returning to country of birth, morbidity patterns, and language skills are but a few of the many factors that are likely to shape experiences of aging and need for health and social care among elderly migrants in Europe [4–9]. The rapid increase in the proportion of elderly migrants has heightened the need to clarify questions of migrants’ perceptions of aging and their potential end-of-life wish to return to their countries of birth.

Anwar [8] proposed ‘the myth of return’ to explain how migrants dream of returning to their countries of birth but in reality often end up staying in the country of immigration. Based on research on first-generation Pakistani immigrants in Britain, Anwar [8] explores how plans for returning to Pakistan were maintained over time. Obligations to relatives in Pakistan and economic and social ties, consisting of flows of remittances, investment abroad, and visits, nourished their wish to return to Pakistan even when their stay in Britain became more permanent following the arrival of the closest relatives. Gardner [9] also finds that elderly migrants glorify their countries of birth as the places they truly belong even though this relationship changes with time, and very few realise the dream of returning.

During an economic boom in the 1960s and 1970s, the Danish state invited workers primarily from Turkey, Pakistan, Morocco, and the former Yugoslavia to Denmark. In 1973, measures were put in place to limit labour immigration, and in the 1980s and 1990s, labour immigration flows were superseded by incoming refugees from Iran, Afghanistan, the former Yugoslavia, Somalia, and Iraq as well as migrants arriving through family reunification. Through family reunification programmes, migrants gradually settled down and became visible in all parts of the Danish system. Immigration patterns thus changed from temporary to ‘temporary permanent’ – a concept indicating a dream of eventually returning to the country of birth [10]. In recent years, new groups of migrants have arrived, mostly from Eastern Europe. Migrants and their descendants now constitute 10.5 % of the Danish population [11], with 15,940 migrants from non-Western countries aged 65 years or over. This number of elderly migrants is set to increase fourfold in the next 20 years [12]. In this study, we focus on the group of aging migrants who arrived predominantly from countries with Muslim majorities. These groups tend to be economically disadvantaged in Denmark and have arrived to Denmark from countries with poor educational systems as well as poor health and social services.

Healthcare services are essential for the increasing elderly migrant population in light of rising morbidity and functional decline. In Denmark, over 80 % of healthcare expenditure is financed by the state – mainly through taxation at the state and municipal level – and is mostly free of charge for all residents. This system aims to provide residents with easy and equal access to healthcare according to need. Access to general practitioners and hospitals is free of charge, though many services for chronically ill elderly people (such as dental services, drugs, physiotherapists, and glasses) require out-of-pocket payments [13]. About 14 % of the expenditures are out-of-pocket payments. Migrants who hold residence permits are covered by the Danish National Health Insurance in the same way as is the rest of the Danish population [14].

Social services for the elderly population in Denmark encompass a range of services delivered at the municipal level in addition to state pensions. Home care is available upon referral and involves support in maintaining activities of daily life, including cleaning, personal hygiene, and intake of medication with the aim of enabling the elderly to remain in the community for as long as possible. For people with significant care needs, homes for the elderly are available. Non-governmental organisations facilitate access to a range of psychosocial services and educational activities such as computer classes, travel, and lectures for the elderly although, to our knowledge, few activities are in place for elderly migrants. A migrant is entitled to receive state pensions if his or her country of birth has a treaty agreement with Denmark. This amount increases with the duration of one’s stay in Denmark, and refugees are entitled to the maximum state pension [15]. Most Danish citizens supplement their state pension with private pensions whereas the income among elderly migrants is often lower due to their shorter inclusion in the Danish labour force.

In Denmark, neither healthcare nor social services have been systematically adapted to the needs of the rising elderly migrant population. Such needs include provision of translators, specific outreach programmes, and culturally adapted services to meet needs among an elderly population representing diverse linguistic, religious, and cultural backgrounds. However, to ensure appropriate services for elderly migrants, we need more insight into perceptions of aging among these groups [1, 6, 7]. The overall aim of this study was thus to explore middle-aged migrant women’s perceptions of aging in Denmark and more specifically the factors shaping their perceptions of whether to remigrate or stay in Denmark during old age.

## Methods

The paper is based on a qualitative study exploring screening behaviours among middle-aged migrant women who have migrated from non-Western societies with Islam as the predominant religion [16]. The study focuses on participation in mammography screening as an example of health behaviour of significant importance due to lower participation rates in mammography screening among migrant women compared to native-born women [17]. A broad approach was taken in order to situate health behaviours within the context of migrant women’s everyday lives in Denmark, including their access to healthcare and social services and their overall quality of life. The study thus aims to acknowledge the interrelationship between health and social factors as well as the importance of taking into account women’s social contexts locally in the country of immigration and extending beyond national borders to their country of birth. One of the issues of concern for the middle-aged women enrolled in the study appears to be adjusting to the prospect of aging and in particular deciding whether to stay in Denmark during the last parts of their lives or returning to their countries of origin. The data underlying this paper thus focuses on the factors related to perceptions of aging that weigh particularly on this decision-making process from the middle-aged migrant women’s perspectives. Since the paper builds upon data collected for a study exploring gender-specific health behaviour (mammography screening), we are unable to include the perspectives of elderly migrant men.

Inclusion criteria for participation in the study were based on age (50–69 years) in order to capture women in the target age groups for the Danish mammography screening programme; country of birth (only including women from non-Western countries known to have low utilisation of this programme); and residence (Copenhagen) to ensure inclusion of women with few access barriers in terms of geographical distance to screening sites.

We recruited women directly and in some cases used gatekeepers from communities with high numbers of migrants, a mosque, and drop-in centres for pensioners or migrants. In addition, snowball sampling was used. By recruiting through existing social networks among the target group, we were able to include women who might otherwise have been less inclined to accept research participation. The final sample of interviewees consisted of 29 women, comprised of women born in Somalia (6), Turkey (5), Pakistan (4), India (2), Iran (1), and Middle Eastern counties with Arabic as the majority language (11). Interviewees differed in terms of duration of residence in Denmark, with some women having arrived rather recently and others being long-term residents (Table 1). Among interviewees, five women were refugees while the rest had migrated to Denmark through family reunification. Due to recruitment in existing social networks and community centres, a few women wishing to attend group interviews were slightly younger/older than the age-range set out in the inclusion criteria. This was the case for five women (ages 47, 48, 49, 78, and 90). We decided to include these women in interviews for ethical reasons and to ensure an appropriate atmosphere for the interviews. The two elderly women were less vocal during interviews, and the perspectives of the three younger women did not surface in discussions related to aging. Statements from these five interviewees therefore do not feature in this paper. In general, interviewees had obtained a rather low level of education (the vast majority had primary school only), and few of them had previous or current employment. Although chronic illness was not part of the inclusion criteria, most interviewees suffered from one or more chronic condition, such as hypertension, diabetes, high blood pressure, heart problems, and arthritis. Most women were Muslims though religious identity was not explicitly discussed during interviews.

**Table 1:** Demographic information

Name	Age^a^	Duration of residence in Denmark^b^	Type of interview
Participant 1	2	3	Individual
Participant 2	2	4	Individual
Participant 3	3	4	Individual
Participant 4	3	3	Individual
Participant 5	3	Missing data	Individual
Participant 6	2	4	Individual
Participant 7	2	2	Individual
Participant 8	2	2	Individual
Participant 9	2	4	Group
Participant 10	3	1	Group
Participant 11	3	4	Group
Participant 12	1	2	Group
Participant 13	2	Missing data	Group
Participant 14	4	4	Group
Participant 15	2	2	Group
Participant 16	2	2	Group
Participant 17	4	1	Group
Participant 18	2	3	Group
Participant 19	3	4	Group
Participant 20	Missing data	4	Group
Participant 21	1	3	Group
Participant 22	2	3	Group
Participant 23	2	4	Group
Participant 24	2	2	Group
Participant 25	1	2	Group
Participant 26	3	2	Group
Participant 27	3	2	Group
Participant 28	3	3	Group
Participant 29	3	3	Group

^a^Age in years: 1 = [40–49], 2 = [50–59], 3 = [60–69], 4 = [70≤]

^b^Duration of stay in years: 1 = [0–9], 2 = [10–19], 3 = [20–29], 4 = [30≤]

Women were informed about the aim of the study as well as research ethics, including confidentiality, storage and use of data, and the right to withdraw at any time during the study. This was explained both orally and in writing in Danish, with interpretation provided as necessary by either professional interpreters or bi-lingual staff/volunteers at the mosque, drop-in centres, or community groups where recruitment took place. Oral informed consent was given by all participants before interviews were conducted. According to official Danish research guidelines, no approval from ethical committees is needed for interview studies. The study was approved by the Danish Data Protection Agency.

Data was collected through a combination of individual semi-structured interviews, focus groups, and informal observation conducted during recruitment and prior to/following interviews. Observations focused on structures and functions of social networks among women; exchanges of tangible and emotional support in these networks; and visible community factors shaping quality of life, including isolation, integration, and insecure neighbourhoods. The combination of two types of interviews was based on our intention of gaining insight into how perceptions of aging and factors influencing these experiences are constructed and negotiated within social relationships. Focus groups gave voice to important dilemmas, for instance regarding the pros and cons of remigration, while a more in-depth understanding of individual life stories was obtained through individual interviews. Each type of interview entails a number of ethical considerations. Focus groups turned out to be very dynamic, with a high degree of discussion and participation although group dynamics at times silenced individual women, who did not seem fully comfortable voicing their opinions, partly due to the risk of breaches of confidentiality. Individual interviews were more intimate and facilitated disclosure of private and sensitive information, e.g. in terms of isolation, illness experiences, and tensions within family relationships. Focus groups were composed based on language group and were facilitated by the researchers. One focus group consisted of women with diverse native languages who met regularly at a community centre and spoke Danish. Each interviewee (expect one) only participated in one type of interview.

Recruitment was relatively lengthy since emphasis was placed on involving women who are otherwise under-represented in health behaviour studies in many European countries due to a combination of lack of familiarity with research engagement and cultural, socioeconomic, and linguistic challenges. Ensuring that potential interviewees were well informed and felt comfortable participating in the study was important for creating a trustful and comfortable interview setting, and it enhanced our insight into the life worlds of women in alignment with the phenomenological starting point for our study. Informal engagement with women, for instance during gatherings at drop-in centres for pensioners and mosques, was thus both a gateway to recruitment and a method of collecting data on social networks, quality of life, and feelings of belonging among these middle-aged migrant women for use as contextual information and in the preparation of interview questions.

The first and the second authors of the present paper conducted eight individual interviews and six focus group interviews in the period from September to November 2011. Challenges in ensuring participation in group interviews forced us to compromise on the number of participants involved in each focus group. This issue arose mainly because some women did not attend the scheduled interview session. We decided to conduct the interviews with the women who did attend the session for ethical reasons although this compromised our ability to achieve the dynamics that were the purpose of choosing this interview type. In total, six group interviews were conducted, each involving 2 to 7 participants. Each interview lasted 30 to 90 min.

The interview guide discussed mammography screening as a part of the larger context of everyday life among migrant women. This included questions concerning perceptions of aging; quality of life and the meaning of social relations; feelings of belonging to Denmark, country of birth, and diaspora communities; experiences with and perceptions of Danish healthcare and social services; and use of healthcare and social services in their country of birth (Table 2). Literacy issues were evident among interviewees, partly due to differences in language skills and little formal education. To overcome language differences, professional interpreters were used in a total of seven interviews. The use of interpreters presented challenges for interaction between interviewer and interviewees, particularly during group interviews [18]. However, all interpreters were trained and had insight into the purpose of the study, qualitative methodology, and research ethics, which helped overcome some of the difficulties associated with use of interpreters in qualitative studies. At the end of each interview, a short questionnaire was handed out with questions about country of birth, number of years in Denmark, morbidity, and household status. Again, due to literacy issues, most interviewees completed the questionnaire orally, with researchers noting down their answers to questions. A few interviewees expressed specific support needs during the interviews (e.g. raised certain health questions or asked for help with getting in contact with specific services), and we did our best to assist them. All interviewees were acknowledged with a gift basket. Interviews were transcribed verbatim in Danish by one of the interviewers. The transcripts from interviews in which an interpreter was involved were authenticated by a second interpreter, who confirmed the correlation between the conversation in the given native language and what was transcribed. This was followed by preliminary analysis of data conducted by two of the authors, who jointly determined the point in the fieldwork process when little new information was produced, after which data collection was concluded.

**Table 2:** Topics discussed during interviews

• Migration history: motives for migrating, experiences during migration and perception of changes in health and social circumstances following arrival in Denmark
• Everyday life in Denmark: social relations to relatives, friends and the wider society, integration and feelings of belonging, roles and responsibilities within families, quality of life, aging expectations, remigration
• Perceptions of health: health status, importance attached to health promotion and early detection, utilization and perceived quality of Danish healthcare services (general practitioner, hospitals and screening programmes) and social services, access barriers (language, knowledge, motivation, social network involvement, geographical distance), perceived risk of diseases
• Perceptions of cancer in general and breast cancer in particular: causes of cancer, severity, perceived vulnerability, family history of cancer, experiences with cancer in country of birth and in Denmark
• Mammography screening: awareness, knowledge of content of invitation and procedure, experiences with participation, barriers for participation and facilitating factors, perceived pros and cons of the programme, suggestions for improvement

### Data analysis and theoretical framework

Data was analysed through multiple readings of the material. The analytical approach was phenomenological, with emphasis on gaining insight into the lived experiences of aging [19]. The attention to immediate experiences and the meanings attached to them helped ensure a data-driven analysis process. Analysis was thus driven by themes emerging as important for experiences of aging. Through initial readings of transcripts, themes related to access to healthcare services, availability of social services, and psychosocial aspects related to social relationships and feelings of belonging emerged. Theories on access to services, social relations, and feelings of belonging were thus used to inform the more detailed level of analysis. Access to healthcare and social services was conceptualised as the actual right to use services and as satisfaction and trust in these services [20]. The concept of social fields provided a method of examining social relations as a set of interlocking networks of social relations through which ideas, practices, and resources are exchanged, organised, and transformed both within nation-states and through transnational ties [21]. Feelings of belonging were conceptualised based on the perceptions that global migration flows create new identity structures in which people feel attached to more than one nation-state and that identity, rather than being confined to one particular nation-state, may span across countries simultaneously [22]. For migrant women, who may have children and grandchildren born and raised in the country of immigration, feelings of belonging may be complex and intrinsically associated with social relations.

Analysis was independently conducted by two researchers and later compared and discussed until agreement was achieved on identified emerging themes, their conceptualisation, and coding of meaning units. To further enhance data analysis, findings were presented and discussed within the research group. Data analysis proceeded through three steps: First, all transcripts were read in their entirety in order to gain an impression of the text as a whole. This was followed by abstraction of major themes related to perceptions of aging and identification of meaning units associated with these themes across the interviews. Finally, the essence of the particular theme was synthesised into a consistent statement across interviews, thereby moving from the concrete to a more abstract level of understanding.

## Results

We identified the following factors shaping perceptions of where to live during old age: 1) importance of access to healthcare services, 2) availability of social services, and 3) social relationships and feelings of belonging. These themes will be described below.

### Importance of access to healthcare services

Co-morbidities and functional decline are increasingly common with old age. Most of the women interviewed had one or more chronic conditions that affected their daily lives and brought them into frequent contact with primary and secondary care.

Ensuring continued access to quality healthcare services was articulated as important for women’s current health status, and they anticipated that this factor would be even more important as they aged. Assessments of need for healthcare services were thus shaped by temporal comparisons with women experiencing declining health at middle age and expecting further decline in upcoming years. In addition, inter-country comparisons were common. Remigration was not desirable as it would entail risks of inappropriate or inaccessible healthcare. In discussions of differences between their current living circumstances and those that would be available to them if they chose to remigrate, interviewees explained how fragmented, low-quality healthcare systems requiring out-of-pocket payments would make it difficult to ensure proper treatments for their chronic health conditions. They thus carefully considered pros and cons of various systems in the light of their morbidity. For some women, inter-country comparisons were based on more recent visits to their countries of origin, through which they had assessed the living conditions available to them should they choose to remigrate. This was primarily the case for those women who had arrived to Denmark through family reunification coming from relatively ‘safe’ countries such as Turkey, Pakistan, Morocco, and India. These women comprised the majority of our sample (17 out of 29 interviewees). In contrast, refugee women from Palestine, Iraq, Iran, Jordan, and Somalia more often referred to media reports, experiences of relatives, or the state of services available prior to their emigration, thereby bringing a temporal dimension into the comparisons. In a group discussion, Participant three outlined the differences between healthcare services in Denmark and India:

There are all those pros and cons on both sides [in relation to staying in Denmark or returning to India]. Over there [in India], we can do the same things as we can do if we fall ill here [in Denmark]. We have to make an appointment, and that takes time, but it [the treatment] is free [here in Denmark]. Over there [in India], on the same day, we would go, pay money, and we get the answer [regarding diagnosis and appropriate treatment]. So, in our country, if we have money… money is very important (…). If I return, then I need to have a lot of money.

The husband of Participant three was ill, and she suffered from chronic diseases. Availability of healthcare services strongly shaped her considerations as to whether she should remigrate to India. While she spoke about her dream of returning to India, financial factors caused her to stay in Denmark in practice.

Regular contacts with general practitioners (GPs) were common among all of the women. Most found their GPs to be easily accessible and had a trusting relationship with him/her, as described by Participant four, who was born in Somalia:

Whenever I go [to the GP], she takes me seriously because she’s been by doctor for almost three years now. So, she informs me about so many things that I should consider [in terms of coping with disease]. Because I have… she has to check me with regards to my liver condition. I have my heart [disease], I have asthma… So, all these complications. I take medicine for the asthma, blood pressure, and I mess up. Sometimes, I don’t know where I’m going. So, when I tell her that, she takes me seriously. She calls me when my blood isn’t… if it’s very high. She sometimes calls me and tells me: “You have to come”. So, she takes me seriously, I’m lucky.

Few women mentioned access problems or perceived inadequate care from their GP, for instance in relation to referral to appropriate secondary care. However, Participant 22, an Iranian woman, described the necessity of actively requesting referrals from her GP: “Something I’ve learned from the beginning is that I don’t go [to the GP] and say: ‘Do you think I should go to the hospital?’ Instead, I say, ‘I’d like you to write that I have to go to the hospital’”.

In discussions about reactions to symptoms, women explained that they felt confident seeking consultation from their GP. Language barriers were rarely mentioned, and access to primary care in Denmark was perceived as similar to that in their countries of birth, apart from the barrier caused by co-payment in their countries of birth. Participant seven from Turkey said that she would engage in the same health-seeking behaviours in Denmark as in her country of birth: “Here, in Denmark, I would, of course, talk to my own doctor, the same as in Turkey. If I suspect [that something is wrong] in Turkey, then I’d see a doctor and talk about it.” Most women did not travel to seek treatment but only used healthcare in their country of birth if needed when visiting relatives.

Experiences with secondary care were mostly associated with inpatient care for chronic conditions and childbirth. The temporal aspect of comparisons was evident here, with women narrating how resources in the healthcare system had worsened in recent years, resulting in poorer quality of care, longer waiting lists for treatment, and shorter hospital recovery periods. Participant three explained: “It isn’t the same treatment anymore (…) Cost savings are happening everywhere now”. Participant three also felt the constraints on referrals to specialist care: “Often, they [GPs] are good but still they won’t help or… I don’t know, it’s like they’re reluctant to provide us with referrals [to secondary care]”.

The overall impression of access to and quality of primary and secondary care was, however, still positive among the women.

### Importance of availability of social services

The vast majority of interviewees were retired or received social welfare benefits (social security or disability benefits) and were thus dependent on social services for their income. In addition, due to their multiple chronic conditions and functional decline, most of the women were in a position in which they needed or soon would need help from others in managing daily tasks such as cleaning and shopping. Therefore, availability of social services was viewed as an additional important factor for deciding where to spend old age.

Most interviewees had children, and in some instances also grandchildren, living in Denmark. They all agreed on the importance of living either with or near their children in order for their children to provide practical and/or emotional support as they grew older and to ensure the maintenance of social roles within extended families. Some women discussed changes in family values and contexts for family life across generations and between countries, noting how their children had established their own families and needed to manage multiple responsibilities in relation to work and family. The women reflected on the strain on these young families and articulated a wish to be able to continue helping the younger generation in their everyday lives despite their transition into old age. Some women also feared that the stressful lives of the younger generation would result in less care for them as they got older. In their discussions about aging and quality of life, some women reflected on the positive aspects they associated with Danish homes for the elderly in particular, mainly since they symbolised publicly funded services that could supplement – or if need be – replace family-based support for the elderly. Alternatives to homes for the elderly, and in particular availability of home care, did not emerge during discussions. Women articulated that social services in Denmark should be adapted to meet the needs of elderly migrants, e.g. in terms of overcoming language barriers and the loneliness that this may lead to among migrants with limited Danish skills. In a group interview, Participant three expressed this need for language-specific homes for the elderly:

I’ve been in England, where they have [homes for the elderly] available in their own language. Like Punjabi, it’s only for Punjabi people, only Punjabi, and only for Indian people. My husband has been there.

Cultural differences and notions of belonging were also perceived as challenges that should be considered in the provision of care for elderly migrants. Participant 22, who had migrated to Denmark from Iran, pointed out the need for homes for the elderly to be adapted specifically to migrant populations whilst acknowledging the difficulties such tailored services could entail:

I think there should be at least one home for elderly for migrants per municipality (…) We understand each other because I’ve seen this [experienced the same things as other migrants], and it helps a bit. I know there’s a fear that such a home could turn into a ghetto.

Although the women in general seemed to have a positive view of Danish social services, some voiced certain reservations about growing old in Denmark, particularly in relation to becoming isolated in homes for the elderly. Women thus articulated how cultural values and social norms surrounding appropriate care for and roles of the elderly perceived to be common in their countries of birth had to be adjusted to the sociocultural context in Denmark, especially when dual responsibilities for younger generations challenged care provided within extended families.

In addition, experiences were shaped by the realm of possibility that women perceived was within their reach. Whereas those women who came to Denmark as voluntary migrants could reflect upon the desirability of remigration, refugee women with little or no opportunity of returning to their countries of birth had little alternative but to come to terms with aging in Denmark. The different issues facing voluntary migrants as opposed to refugees is illustrated by the following quote articulated by Participant 13, a Palestinian refugee woman, during a group discussion of the pros and cons of social care in Denmark versus their countries of birth: “You’d prefer to die in your own country rather than dying in a foreign country (…) And no matter where you live and how well you’re feeling in another country, it’s always more pleasant and better to return to and die in your own country”.

Participant 13 describes herself as a foreigner in Denmark and feels a sense of belonging to her country of birth. A dream of returning thus still existed, yet for many of the women who came to Denmark as refugees from Palestine, Somalia, or Iran, it was impossible to return due to security, legal, and financial issues. Participant five, a woman from Somalia, explained how she cared for her paralysed ex-husband in Denmark, hoping he could repatriate to Somalia. She was in doubt as to whether she could return to Somalia herself since she had a child and grandchildren in England and family to support financially in Somalia through her Danish pension:

I’d like to help him [the ex-husband]. Take him to Somalia when he gets permission to repatriate. [Interviewer: But you wouldn’t like to stay there [in Somalia]?]. First, let’s see how he’s feeling. If I think it’s okay, then I’ll do it [stay in Somalia]. If it turns out to be fine, then I want to… If it’s possible that way.

Interviewees who were not refugees often attempted to combine living in Denmark with longer stays in their countries of birth, mostly through regular travel, but due to restrictions on stays abroad by people receiving social benefits, this was often hard to accomplish. A woman from Turkey explained how she would prefer to stay six months in each country annually but was prevented from doing so due to such legislation.

Most women wished to stay in Denmark since this would enable their children and grandchildren to take care of them and since this opened up the possibility of accessing support from Danish social services if needed. However, a few women, most of whom were refugees, mentioned their desire to die in their countries of birth.

### Emphasis given to social relationships and feelings of belonging

The women’s social relationships in Denmark and a feeling of belonging had over time created a desire to stay in the country during old age. The geographical location of their husbands and children seemed particularly important for their decisions to stay in Denmark. In a group interview, Participant 15, who was born in Iraq, spoke about her possibilities of returning to Iraq: “Because we have children here, my daughter here, and all family here, what should I do in Iraq? Only my mother [is in Iraq]. My mother is an old lady, and my sister. But all family is here. I can’t leave”.

Temporality again surfaced in the women’s accounts. They explained how, in the process of settling down in Denmark, they had gradually become embedded in social relations, and relatives and friends in their countries of birth were gradually accorded less importance over time.

The issue of returning during old age was an ongoing dilemma in the women’s lives. It was clearly a topic they had discussed with each other before, with the result that they actively spoke about the dilemmas during the interviews. Few women appeared determined not to return. As Participant 22, an Iranian woman, explained: “I’ve fled [the country], and I’ll never return. So for me, that’s clear.” She had witnessed the killings of her father and brother and had fled to Denmark alone and had since then focused on creating a social network in Denmark.

The majority of the women had also established social lives in Denmark, with important social relationships with other migrants living in the country. Social relationships with women living with similar dilemmas pertaining to feelings of belonging and pros and cons of returning to countries of birth were clearly important sources of support and understanding. These relationships also influenced their desire to stay in the country.

The women who came to Denmark for family reunification and had their closest relatives in the country generally expressed a wish to stay in Denmark, but questions of returning to their countries of birth during old age still seemed to be a point of discussion among them. Feelings of belonging in Denmark, however, were naturally influenced by the amount of time spent in the country and their reasons for migrating. Expressions of identity phrased as “feeling Danish or partly Danish” were common among the women with a longer duration of stay in Denmark. Most of these women had migrated to the country for family reunification and found it difficult to imagine returning to their countries of birth, partly due to having undergone significant changes themselves since emigration and partly due to changes that had occurred in their countries of birth. Participant 23 from Pakistan, who had lived in Denmark for almost 40 years, explained: “I feel more Danish in my behaviour and thinking [the way I think] and such things, but Pakistan is still inside of me. But when we travel to Pakistan for a month and up to a month and a half, then you get tired, then you would like to go home [to Denmark]”.

Others, mostly refugees, who had spent much less time in the Denmark, often said that they still felt strongly connected to their countries of birth. Concurrently, the women described how they valued the democracy and feeling of safety in Denmark, pointing out that discussions on returning were an ongoing dilemma. Inter-country comparisons were obvious as these women came from politically unstable countries with extreme poverty, and Denmark had become a safe heaven. Return migration was often not possible for these women due to economic difficulties and lack of security in their countries of birth. Participant one, a Palestinian woman who fled from Jordan, explained:

For me, I’m happy to be here. Especially, as I told you, when I came to Denmark, we feel free. Here, we can talk… We can do what we want. I mean, especially, we were forbidden [in country of birth], afraid to talk about everything. (…) Now it’s too late to [return]. I feel like, this country, I’ve got used to it. I came here when I was nearly 30, 32, something like that. Now, I’m nearly 60, so I’ve got used to it (…).

Participant one articulated how she no longer felt she belonged to one specific geographical location and how she felt she could not identify with a specific nationality:

I’m half Syrian, half Jordanian, or half Syrian, half Palestinian, and I feel with the

Syrian that I’m Syrian, I feel with the Palestinian that I’m Palestinian. But at the same time, I just like to be Muslim. All nationalities, for me, I don’t like it, I don’t feel it. Of course, I have a tradition with foods and all that, but I love Muslims, I love them, really, I love them. Of all nationalities, whatever they are, I love them.

Throughout their time in Denmark, the women had settled down with families and friends and had come to an understanding that the immigration country was a place of permanent residence rather than a temporary solution meeting the needs for security and prosperity that had been the causes of migration. Women thus articulated their desire to stay in Denmark during old age although they simultaneously felt attached to other geographical locations.

## Discussion

Among this sample of middle-aged migrant women, most of whom suffered from chronic disease, perceptions of aging and attitudes towards remigration were shaped by the importance of easily accessible and high-quality healthcare services as well as the availability of social services perceived as being needed during old age. Social relationships and feelings of belonging in the country of immigration further informed the decision not to remigrate, and the women held positive views overall on growing old in Denmark. However, remigration seemed to be a subject of ongoing discussion among these women, with some expressing ‘a dream’ of returning to their countries of birth during old age, thereby supporting the findings made by Anwar [8]. Since only a few of the interviewees had reached old age, it is important to acknowledge that perceptions of where to live during the final part of one’s life were, for most women, still imaginary rather than actual dilemmas that needed to be confronted. The rather open and positive attitude to homes for the elderly may partially be explained by this age effect. Importantly, perceptions of where to spent one’s old age were shaped by migrant status, with refugees facing constraints that made it difficult to even envision aging in their countries of birth.

Access to healthcare services is related to both primary and secondary care. Due to increasing morbidity, the interviewees had frequent health assessments and referrals to specialist care made by their GPs. They expressed trust and satisfaction with their GPs, which may seem surprising in context of the language barriers experienced by most of the women, which may impede access to and quality of healthcare delivery [23]. These positive perceptions perhaps point to the importance of the primary healthcare structure in Denmark in which every citizen is listed with a community-based GP, who serves as an entry point to diagnostics, treatment, and specialised care for many age-related health conditions. As a result, many patients form long-term relationships with their GPs, thus partly explaining the high levels of trust and satisfaction that women voice in discussions about their utilisation of primary care.

In terms of the quality of secondary, hospital-based care, women expressed more mixed perceptions but still preferred Danish healthcare services, which were perceived to be based on need rather than ability to pay, in contrast the situation in most of the women’s countries of birth. In Denmark, most patient satisfaction surveys are conducted in Danish and do not include large numbers of ethnic minorities. A number of studies among migrants in Europe point to negative perceptions of accessibility and quality of healthcare due to language barriers, differences in expectations, and perceived discrimination, combined with reluctance to assert healthcare rights among [24, 25]. However, particularly at an age with increasing morbidity and functional decline, such healthcare services may be preferable to those of low- or middle-income countries.

As for social services, it seemed that interviewees held surprisingly positive views concerning homes for the elderly, although it is difficult to judge from the interviews whether the women would actually use them or preferred care provided within family networks. Most of the interviewed women had migrated from countries where there are markedly different expectations as to the social roles for elderly people as well as different social norms regarding provision of care for the elderly. These expectations were acknowledged as difficult to meet in the Danish context, and most women thus seemed to accept new forms of care, such as homes for the elderly, to supplement or replace care provided by relatives. Although cultural values may suggest that elderly migrants have high expectations for family support and will be reluctant to use formal sources of elderly care, several qualitative studies among older migrants in Britain suggest that elderly migrants acknowledge the difficulty of upholding the ideal of family support whilst also accepting and expecting high standards of social services [6, 7]. Nevertheless, some women voiced concerns about Danish social services, in particular in terms of risks of isolation and language barriers. This finding is supported by a study by Mian [26], who identified very few Pakistani elderly women at homes for the elderly in Denmark, mainly caused by perceived risk of language barriers, fear of losing contact with their children, and a perception of homes for the elderly as an unfamiliar concept. The establishment of separate sections within homes for the elderly dedicated to migrants, who may share experiences of migrating from one country to another, could create a safe environment, yet results from the present study indicate the problems with establishing such homes, particularly in terms of the lack of a shared cultural and linguistic background across diverse migrant groups. Interestingly, other social services for the elderly, such as home care, were not mentioned as alternatives to family-based care. This could be due to limited awareness of these forms of care since studies among older migrants in Britain indicate that these migrants lack knowledge of social services available to the elderly [6, 7].

Social relationships with children, grandchildren, husbands, and other migrant women living in Denmark also influenced the women’s quality of life with respect to aging and return migration. All of the women had transnational ties to relatives in their countries of birth [21, 27], but those women who came to Denmark for family reunification a long time ago had spent many years in Denmark and had often established family and extended social networks in the country. These women did not articulate plans of returning to their countries of birth. For the women who arrived to Denmark later on as refugees, additional factors seemed to influence their experiences with aging in Denmark, and there seemed to be a tendency for these women to miss their country of birth more. It is worth noting that the Danish state offers financial support for refugees who choose to leave the country – a benefit that could influence decisions on returning to their countries of birth. However, the women in the present study stressed the importance for their quality of life of well-functioning healthcare and social services in Denmark and the ability to stay with their children and husbands. A Danish study has likewise shown the importance of social relationships with children and grandchildren among elderly Turkish and Pakistani immigrants in Denmark, suggesting that remigration depended upon whether younger generations were willing and able to leave Denmark [10]. The former study and our data, however, indicate that the children and grandchildren of middle-aged and elderly migrants seem to settle down in Denmark and do not wish to return to a country in which they will lack social networks, experience fewer job opportunities, and have access to substandard education systems compared to the Danish system. War and poverty in their countries of birth additionally made it difficult for the women to return, and the women expressed concerns about having become estranged from the country since it may have changed dramatically while they were away. The women seemed to long for their countries of birth as they were before they fled. The women thus had conflicting desires: They longed for their countries of birth but at the same time did not wish to return. After having lived in Denmark for many years, the women in the present study had become accustomed to new ways of life, and they worried that, if they returned to their countries of birth, it would be difficult for them to adjust to life in countries they had left many years earlier. At the same time, many of the women were the primary provider for relatives in their countries of birth through remittances and, as such, were dependent on their state pension in Denmark. Decision-making with regard to preferred place of aging therefore emerged as a complex process involving the balancing of the various personal and social circumstances influencing these middle-aged migrant women.

Within the health sciences literature, the so-called ‘myth of return’ is maintained, suggesting that there is little need for adaptation of healthcare and social services to accommodate the needs of elderly migrants as these groups are likely to remigrate after retirement [4, 8]. However, the present results concur with previous studies indicating that even though migrants might dream of returning to their countries of birth, they seem to prefer staying in the country of immigration in practice [8, 9]. Among the middle-aged women in the present study, accessible healthcare and social services, relationships to their closest family, and feelings of belonging in Denmark nourished a wish to stay rather than remigrate. Nevertheless, our data underscores that decision-making processes are complex and shaped by a number of situational factors, in particular for refugee women, who are prevented from remigration by legislation, security concerns, and/or responsibilities for sending remittances to relatives in their countries of birth. As we recruited interviewees through diverse communities for migrants and were able to conduct interviews within existing social relationships, women seemed to be more easily recruited and more comfortable sharing experiences and perceptions related to aging. We were able to recruit women with little formal education who also faced language barriers, socioeconomic vulnerabilities, and suffered from one or more illnesses. These women are often underrepresented in migrant studies, and in several instances, they voiced appreciation of having the opportunity to discuss their perceptions and needs for support in achieving as good a quality of life as possible during old age. As such, findings are likely to tap into perceptions among low-literacy migrant women. Nevertheless, women included in our study were not the most isolated since they engaged with other migrants and seemed comfortable critically discussing their perceptions of living as migrant women in Denmark and the associated dilemmas related to aging. More isolated women would likely face additional challenges in coming to terms with aging-related dilemmas and choices. The high prevalence of chronic illness among our sample has important implications for findings and may in particular explain the importance given to accessibility and quality of healthcare services in Denmark. In addition, the emphasis placed on healthcare services during interviews may be shaped by the fact that women were recruited for a study on mammography screening, thus likely setting an agenda focused on disease and healthcare access. We tried to counter this by emphasising broader aspects related to aging, including well-being, social networks and quality of life. Although aging is of course associated with risks for declining functional ability and rising morbidity, for healthier middle-aged women, choices of returning to their countries of birth are likely less dependent upon accessibility and availability of healthcare services. Since this study is cross-sectional, we are unable to explore how perceptions change over time and how factors such as illness, death of a spouse, or intergenerational dynamics may shape perceptions of remigration during old age. From our data, questions of returning to their countries of birth seemed to be a topic frequently debated within social networks, and decision-making developed over time. Longitudinal qualitative studies are needed to explore the dynamics of this process and how it is shaped in response to, for instance, contextual factors in Denmark and countries of birth. Last but not least, qualitative studies among migrant men are warranted to further our understanding of the experiences of aging among these population groups.

The results pave the way for discussion of how to think about ‘home’ in a globalised world. In a time of globalisation, ‘home’ is no longer a fixed place but has increasingly turned into something fluid; a set of practices, memories, and myths [9]. Therefore, as Gardner [9] argues, there is no “easy return” for migrants. The maintenance of transnational ties along with a desirable life in Denmark resulted in the women often expressing feelings of belonging to more than one geographical location. As argued by many migration scholars, migration cannot be understood as a one-way movement that results in the gradual integration of migrants into the receiving country [21, 27]. While migration by definition means a change in geographical location, migrants retain ties to their countries of birth as well as to relatives settling down in other countries. Integration into a country and enduring transnational ties are therefore neither incompatible nor binary opposites [27]. Migrants might feel that they belong to a variety of geographical locations simultaneously. In our study, the women voiced feelings of belonging to both Denmark and their countries of birth, in many ways becoming citizens of the world through their global pathways and networks [21]. The women were satisfied with the quality of life in Denmark and wished to stay in the country. Healthcare and social services should therefore embrace the needs of the growing number of elderly migrants, some of whom, particularly refugee women, may experience language barriers, fragile social networks, and significant responsibilities and emotional ties to their countries of birth, which must be taken into account in service-delivery [4, 6, 7, 28]. Some needs might be met by securing access to translators as well as conducting a systematic assessment of social relations followed by a referral to, e.g., volunteers and organisations for elderly people. In addition, separate sections dedicated to migrants at homes for the elderly could be considered in order to create a culturally and linguistically familiar environment for the elderly, although these could be difficult to implement due to the diversity among these groups and could lead to segregation from other ethnic groups.

### Limitations of the study

Due to the relatively limited data sample covering diverse groups of migrants, the present study is explorative and particularly points to the need for longitudinal qualitative studies among larger groups of migrant women in order to understand the needs of populations with diverse ethnic and social backgrounds. As the present analysis builds upon data collected as part of a broader study on health behaviour among migrant women, the depth of data collected in relation to each of the themes covered in the analysis does not allow for a more elaborate, theory-driven analysis exploring each theme in greater detail. More in-depth qualitative studies, preferably employing a longitudinal design following women as they age, will be necessary to ensure a better understanding of the complexities of aging in a European migration context. A longitudinal design would furthermore enable identification of the extent of future remigration among these middle-aged women.

Women who are in a positive financial situation and who originally migrated from more affluent countries with well-developed health care systems probably face fewer difficulties travelling as well as paying for and using healthcare services in their countries of birth compared with the women included in our study. Perceptions of aging and quality of life may also differ between genders, and there is a need for more studies among migrant men. In addition, studies are needed to identify specific needs in encounters between middle-aged and elderly migrants within the context of current healthcare and social services.

## Conclusions

Migrants’ perceptions of aging, including whether to remigrate or stay in the immigration country, are shaped by multiple factors, including access to quality healthcare and social services, social relationships, and feelings of belonging. The women represented in the present study emphasised the importance of access to the Danish healthcare system and held positive views on social care available to the elderly in Denmark. Social relationships in Denmark, most importantly intergenerational ties, further nourished their desire to stay in the country although some women, especially those who came as refugees, dreamed of returning to their countries of birth during old age. Fear of having been estranged from values and practices in their countries of birth as well as financial and safety issues kept them from doing so. Our findings suggest that elderly migrants from diverse cultural and linguistic backgrounds will choose not to remigrate and will instead utilise healthcare and social services in Denmark. This necessitates continued focus on ensuring that needs among Europe’s increasing elderly migrant population are adequately met.

## Abbreviation

GP: General practitioner
